# Spatial and Temporal Characteristics of Environmental Air Quality and Its Relationship with Seasonal Climatic Conditions in Eastern China during 2015–2018

**DOI:** 10.3390/ijerph18094524

**Published:** 2021-04-24

**Authors:** Zhiyuan Wang, Xiaoyi Shi, Chunhua Pan, Sisi Wang

**Affiliations:** College of Geography and Environmental Sciences, Zhejiang Normal University, Jinhua 321004, China; xiaoyi.shi@zjnu.edu.cn (X.S.); 202020200740@zjnu.edu.cn (C.P.); wangsisi@zjnu.edu.cn (S.W.)

**Keywords:** environmental air quality, air quality index, eastern China, spatial and temporal characteristics, climatic conditions

## Abstract

Exploring the relationship between environmental air quality (EAQ) and climatic conditions on a large scale can help better understand the main distribution characteristics and the mechanisms of EAQ in China, which is significant for the implementation of policies of joint prevention and control of regional air pollution. In this study, we used the concentrations of six conventional air pollutants, i.e., carbon monoxide (CO), sulfur dioxide (SO_2_), nitrogen dioxide (NO_2_), fine particulate matter (PM_2.5_), coarse particulate matter (PM_10_), and ozone (O_3_), derived from about 1300 monitoring sites in eastern China (EC) from January 2015 to December 2018. Exploiting the grading concentration limit (GB3095-2012) of various pollutants in China, we also calculated the monthly average air quality index (AQI) in EC. The results show that, generally, the EAQ has improved in all seasons in EC from 2015 to 2018. In particular, the concentrations of conventional air pollutants, such as CO, SO_2_, and NO_2_, have been decreasing year by year. However, the concentrations of particulate matter, such as PM_2.5_ and PM_10_, have changed little, and the O_3_ concentration increased from 2015 to 2018. Empirical mode decomposition (EOF) was used to analyze the major patterns of AQI in EC. The first mode (EOF1) was characterized by a uniform structure in AQI over EC. These phenomena are due to the precipitation variability associated with the East Asian summer monsoon (EASM), referred to as the “summer–winter” pattern. The second EOF mode (EOF2) showed that the AQI over EC is a north–south dipole pattern, which is bound by the Qinling Mountains and Huaihe River (about 35° N). The EOF2 is mainly caused by seasonal variations of the mixed concentration of PM_2.5_ and O_3_. Associated with EOF2, the Mongolia–Siberian High influences the AQI variation over northern EC by dominating the low-level winds (10 m and 850 hPa) in autumn and winter, and precipitation affects the AQI variation over southern EC in spring and summer.

## 1. Introduction

The World Health Organization (WHO) has stated that over 90% of the world’s population are affected by high levels of air pollution, and nearly 7 million premature deaths related to air pollution occur each year [1]. China is one of the most severely air polluted countries in the world [2,3], which may significantly impact the health of its citizens [4,5,6,7] and economic development [8,9]. Therefore, atmospheric contamination has attracted the attention of the Chinese public and government, and become one of the most important environmental issues in current societies [10].

Environmental air quality (EAQ) is mainly affected by the combined effects of human activities and climatic conditions [11]. On a regional scale, the emission of air pollutants is a significant contributor to ambient air pollution [12]. In the past decade, due to the continuous expansion of Chinese intensive industry and the increase in production capacity and energy consumption, EAQ has deteriorated severely [13]. The levels of primary and secondary air pollutants are serious in some heavily industrialized and densely populated regions, such as the northern plains of China [6,14,15], Northwest region [16], Yangtze River Delta [17], and Pearl River Delta [18]. The people in these regions are exposed to high levels of air pollution. Although new energy-saving and emission control policies can effectively improve the EAQ in some regions [19,20,21], on a large scale, the spreading and migration of air pollutants are inevitably influenced by regional or even global climatic conditions [22]. Studies have highlighted that the emission of air pollutants in some regions of China has decreased significantly in recent years, but the regional air quality index (AQI) remains high, and air pollutant emissions from surrounding regions are the major source of local atmospheric contamination [23]. Scholars have proposed a policy of “joint prevention and control of regional air pollution” [24,25], suggesting that understanding the principles of air pollutant spreading and migration is an effective way of solving regional ambient air pollution problems. The climatic/meteorological condition is an important factor in understanding the spread and migration of ambient air pollutants.

Recently, with the accumulation of basic research data and the continuous progress of technology, the research on the relationship between EAQ and climatic/meteorological conditions in China has achieved fruitful results. Studies based on continuous on-site monitoring data have explored the characteristics of regional EAQ and the response to different climatic/meteorological conditions in China [26,27,28]. However, due to the limitations of the data collection system, the published studies on EAQ variations have focused on Chinese modern economic and cultural development centers [29], such as Beijing [19,30], Shanghai [31], and Guangzhou [32], and on short time-scales of a few days or weeks. Relatively few studies have been conducted on the relationship between large-scale climate circulation and EAQ in the longer term. With the rise of remote sensing technology, the use of remote sensing data to estimate long-term EAQ across a large area has attracted growing efforts [9,33,34]. However, satellite data are of limited utility at present. Some data limitations, such as inaccurate retrieval algorithms, the interference of cloud and snow, the discontinuity of observation data, and the inversion accuracy of aerosol optical thickness, can influence measurements and result in inaccuracies [35]. Therefore, to investigate the association between long-term climate conditions and EAQ in China, large-scale and long-term monitoring datasets have an important role to play.

Environmental monitoring is the fundamental means of understanding, grasping, evaluating, and predicting EAQ. Continuous monitoring data of EAQ could scientifically evaluate EAQ status, and provide a reference for the development of atmospheric environmental protection measures. In recent years, with the rapid development of China’s social economy, the Chinese government has established a national EAQ monitoring network. In February 2012, the Ministry of Environmental Protection (MEP) of China approved a new National Ambient Air Quality Standard (BG3095-12) to monitor real-time ground-level air pollutants. From January 2013 onward, environmental monitoring stations in more than 100 Chinese cities began to release environmental monitoring data, including the concentrations of six major air pollutants, i.e., carbon monoxide (CO), sulfur dioxide (SO_2_), nitrogen dioxide (NO_2_), fine particulate matter (PM_2.5_), coarse particulate matter (PM_10_), and ozone (O_3_), making it possible to study the large-scale characteristics of EAQ and its relationship with the background climate in China. For the first time, Wang et al. [36] examined the spatial and temporal variations of major air pollutants derived from 286 monitoring stations in 31 cities in China, from March 2013 to February 2014, and found that PM_2.5_ was the primary pollutant that affected EAQ in 2013. Based on monitoring data in 31 provincial capital cities in China between April 2014 and March 2015, Zhao et al. [37] found that the concentration of particulate matters (PM_10_ and PM_2.5_) was significantly higher in winter than in summer, whereas O_3_ had the opposite distribution. The concentrations of SO_2_ and CO in the cities of northern China were significantly higher than those of southern China in 2014. Moreover, urban air pollution had a significant spatial aggregation or clustering, that is, there was a close relationship between urban air pollution and that of the neighboring cities [38]. Previous research found that relative humidity was negatively associated with the EAQ in the northern plains of China in the autumn and winter of 2013 [39]. Compared with 2014, the climatic/meteorological conditions in 2015 were adverse for the spreading of air pollutants in some regions in China [40]. Furthermore, the frequency and duration of severe haze weather events in northern China were also related to specific climatic conditions in winter [41]. The rapid decline in the extent of Arctic sea-ice, which is caused by global warming, and reduced precipitation and surface winds could have intensified the haze pollution in eastern China [42], which can explain the 45%–67% of interannual to decadal haze pollution variabilities after 2000 [43]. Hence, climatic conditions may play a non-negligible role in influencing the EAQ on a large scale.

The above studies explored the spatial distribution of EAQ in major Chinese cities in recent years and its relationship with climatic/meteorological conditions, providing important theoretical references and practical recommendations for this study. However, due to the monitoring data of EAQ being sparse in time and space, the major spatial and temporal distribution of EAQ in China and its response to climatic conditions remain less understood. Therefore, the aim of this study was to understand the major spatial and temporal variations of AQI, using 4-year (January 2015–December 2018) EAQ data released by the MEP for nearly 1300 monitoring stations in Eastern China (EC), and their associations with climatic conditions, hoping to provide a scientific reference for Chinese EAQ governance.

## 2. Data and Methods

### 2.1. Data Sources

The hourly air pollution data of SO_2_, NO_2_, PM_10_, PM_2.5_, CO, and O_3_ for 1615 environmental monitoring stations between January 2015 and December 2018 were analyzed to assess EAQ in China. Real-time hourly concentrations of air pollution data were downloaded from the National Environmental Monitoring Center (http://113.108.142.147:20,035/emcpublish/, accessed on 1 February 2021). At each station, automated monitoring systems were installed and used to measure the concentration of SO_2_, NO_2_, CO, and O_3_ according to China Environmental Protection Standards HJ 193-2013 (http://www.es.org.cn/download/2013/7-2/2627-1.pdf, accessed on 5 February 2021), and of particulate matter according to China Environmental Protection Standards HJ 655-2013 (http://www.es.org.cn/download/2013/7-12/2626-1.pdf, accessed on 5 February 2021).

We acquired the climate data from the ERA-interim (ERAI, https://www.ecmwf.int/en/forecasts/datasets/reanalysis-datasets/era-interim, accessed on 1 May 2019) reanalysis data of the European Center for Medium-Range Weather Forecast (ECMWF), which is available monthly. The ERAI reanalysis has improved significantly the global atmospheric records of mass, moisture, energy, and angular momentum compared with ERA-40 [44]. Compared with other reanalysis data, ERAI reanalysis products are closer to the real observation data of China, in climatic elements such as temperature, atmospheric circulations, and cloud water distribution [45,46,47]. These efforts ensure the trustworthiness of the conclusions made in this study. Given the main objectives of this study, the spatial resolution of climatic elements derived from the ERAI was 0.5° × 0.5°, and the period was from January 2015 to December 2018, the same as the air pollution data (more information in Table 1).

### 2.2. Methods

**Figure 1:**
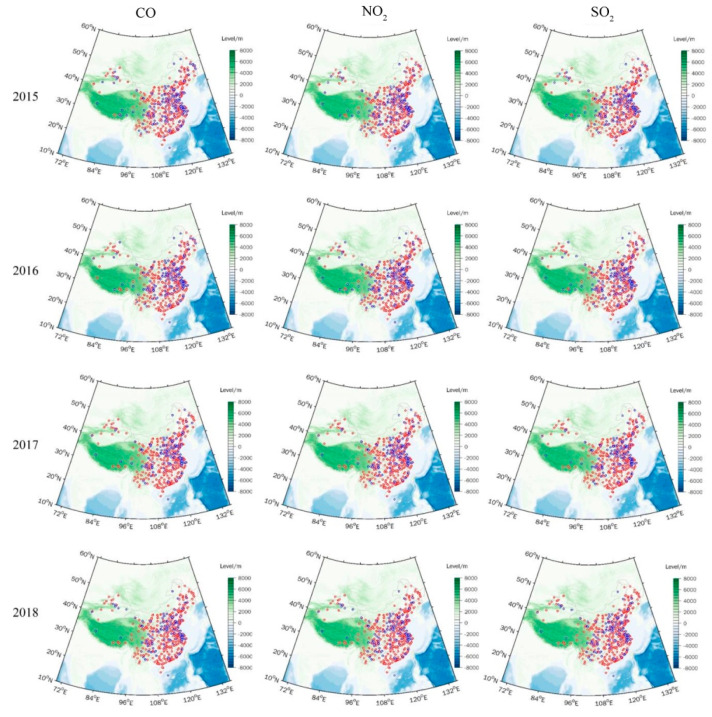

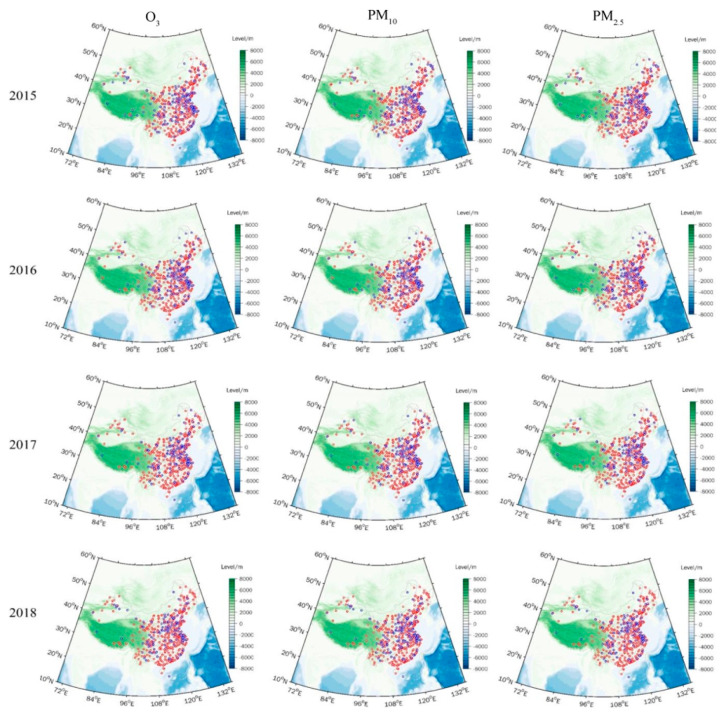
Locations of effective monitoring stations of CO, NO_2_, SO_2_, 8 h O_3_, PM_10_, and PM_2.5_ in China during 2015–2018 (except Hong Kong, Taiwan, and Macao). The blue dots indicate locations of invalid monitoring stations (number of missing days/total number of days in the year ≥5%); the red dots indicate locations of effective monitoring stations (number of missing days/total number of days in the year <5%).

According with the aims of the study, the original air pollution monitoring data derived from the stations was screened and processed. First, we computed daily averages by averaging hourly CO, SO_2_, NO_2_, PM_2.5_, and PM_10_, and maximum average 8 h O_3_ values in a day. Missing values in the data were replaced by linear interpolation. For a certain type of monitoring data, if the number was less than eight points in a day, it was considered as the missing value for that day. Second, we calculated the total missing days for the six types of air pollutant during 2015 to 2018. For each monitoring station, if the number of missing days for a type of air pollutant was less than 5% of the year, it would be classified as an “effective monitoring station”. Otherwise, the station was classified as an “invalid monitoring station”. The locations of the effective (red dot) and invalid (blue dot) monitoring stations during 2015 to 2018 are shown in Figure 1. The number of effective monitoring stations for each air pollutant for every year was greater than 1300 (the total number of monitoring stations is 1615), accounting for more than 80% (Table 2), and covering 369 Chinese cites by statistical calculations. To ensure the reliability of the study results, air pollutant data derived from invalid monitoring stations each year were removed in this study.

Due to the most effective monitoring stations being located in EC (Figure 1), this study mainly focused on characteristics of EAQ in EC (105–135° E, 20–55° N). To better explore the major spatial and temporal characteristics of monthly AQI, as well as its relationship with climate conditions in EC during 2015–2018, the data were processed as follows: (1) Interpolation of air pollutants data (daily mean derived from the effective monitoring stations during 2015–2018) to a regular 0.5° × 0.5° grid using the cubic spline method, which was the same as the spatial resolution of the climate data covering the EC; (2) calculation of daily average AQI using interpolated air pollutant data for each grid point of EC, referring to Chinese ambient air quality standards (GB3095-2012, https://www.mee.gov.cn/ywgz/fgbz/bz/bzwb/dqhjbh/dqhjzlbz/201203/W020120410330232398521.pdf, accessed on 5 February 2021) issued by CMEP. The calculation formula of AQI is:

$$\mathrm{AQI}=\max\left({\mathrm{IAQI}}_{{\mathrm{SO}}_{2}},{\mathrm{IAQI}}_{{\mathrm{NO}}_{2}},{\mathrm{IAQI}}_{\mathrm{CO}},{\mathrm{IAQI}}_{{\mathrm{PM}}_{2.5}},{\mathrm{IAQI}}_{{\mathrm{PM}}_{10}},{\mathrm{IAQI}}_{O_{3}}\right)$$

$${\mathrm{IAQI}}_{p}=\frac{{\mathrm{IAQI}}_{\mathrm{Hi}}-{\mathrm{IAQI}}_{\mathrm{Lo}}}{{\mathrm{BP}}_{\mathrm{Hi}}-{\mathrm{BP}}_{\mathrm{Lo}}}\left(C_{P}-{\mathrm{BP}}_{\mathrm{Lo}}\right)+{\mathrm{IAQI}}_{\mathrm{Lo}}$$

where p represents the pollutant p (SO_2_, NO_2_, CO, PM_2.5_, PM_10_, and O_3_); ${\mathrm{IAQI}}_{p}$ represents the individual AQI (IAQI); $C_{P}$ is the measured concentration of p; ${\mathrm{BP}}_{\mathrm{Hi}}$ represents the high threshold of $C_{P}$ interval; ${\mathrm{BP}}_{\mathrm{LO}}$ is the low threshold of $C_{P}$ interval; ${\mathrm{IAQI}}_{\mathrm{Hi}}$ is the IAQI values of ${\mathrm{BP}}_{\mathrm{Hi}}$; and ${\mathrm{IAQI}}_{\mathrm{Lo}}$ is the IAQI values of ${\mathrm{BP}}_{\mathrm{LO}}$. The maximum ${\mathrm{IAQI}}_{p}$ of all pollutants is chosen as the overall AQI. According to the stage of development of China’s socio-economic conditions, the IAQI threshold interval was set as the medium-term target recommended by the WHO (Table 3). (3) The monthly AQI was simply calculated by averaging the non-missing daily AQI for the month. 

To obtain the major spatial and temporal characteristics of AQI distribution in EC during 2015–2018, one technique used for such studies is to decompose the spatial AQI of EC into patterns called empirical orthogonal functions (EOF), and these patterns are ranked according to how much of the total variance they explain. Each EOF is multiplied by a time-dependent coefficient, which is called the principal component (PC) of that EOF. Often a few of these EOFs with their PCs explain most of the variability, and in some cases a particular PC has a temporal fingerprint that matches the climatic signal. EOF has been widely used in meteorology to analyze various meteorological elements. Its fundamental function is decomposing the variable matrix $X_{i\times j}$, which consists of the AQI of j times (time scale of samples) at i spatial points, into the linear combination of a spatial eigenvectors matrix V and their associated time coefficients matrix T:

$$X_{i\times j}=\left[\begin{array}{ccc} V_{11} & \cdots & V_{1i}\\ \vdots & \ddots & \vdots \\ V_{i1} & \cdots & V_{\mathrm{ii}} \end{array}\right]\left[\begin{array}{ccc} T_{11} & \cdots & T_{1j}\\ \vdots & \ddots & \vdots \\ T_{i1} & \cdots & T_{\mathrm{ij}} \end{array}\right]$$

For nth AQI value $x_{\mathrm{mn}}$ at mth point, EOF expansion is to decompose $x_{\mathrm{mn}}$ into the sum of products of spatial functions and temporal functions:

$$x_{\mathrm{mn}}=\overset{i}{\underset{k=1}{\sum }}V_{\mathrm{mk}}T_{\mathrm{kn}}=V_{m1}T_{1n}+V_{m2}T_{2n}+\cdots +V_{\mathrm{mi}}T_{\mathrm{in}}$$

The eigenvector characterizes the spatial pattern of a reginal AQI field. The associated time coefficients delineate the temporal variation character of the spatial pattern characterized by the eigenvector. A positive associated time coefficient indicates that the pattern is the major trend of a variable at that moment; conversely, a negative associated time coefficient indicates that the variable displays an opposite trend of the pattern; and the larger the absolute value of the associated time coefficient, the more significant the associated spatial pattern. For more information about EOF method, we refer to the study by North et al. [48].

If not specified otherwise, here, spring means March–April–May, summer means June–July–August, autumn means September–October–November, and winter means December–January–February.

## 3. Spatial and Temporal Characteristics of AQI

**Figure 2:**
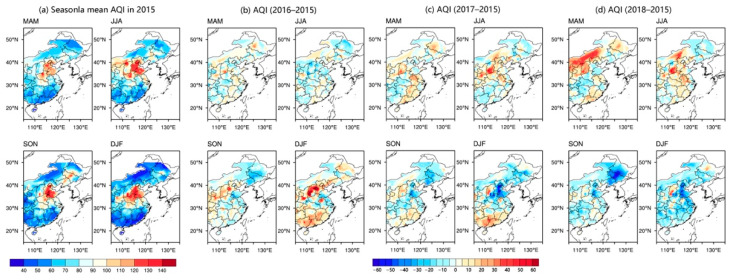
Spatial distributions of seasonal mean AQI in 2015 (**a**), and the differences of corresponding seasons with the years 2016 (**b**), 2017 (**c**), and 2018 (**d**), respectively. MAM indicates spring, JJA indicates summer, SON indicates autumn, DJF indicates winter.

The spatial patterns of the seasonal average AQI in EC in 2015 are shown in Figure 2a. Generally, the EAQ was significantly better than other seasons in summer, and worst in winter in EC in 2015. The regional average concentrations of the six air pollutants all reached their lowest in summer and the highest in winter (with the exception of O_3_). The Asian monsoon system, which brings more precipitation to EC in summer, offers increased air purification capacity. Therefore, the AQI was relatively low in summer (Figure 2a). Starting in the autumn, using coal for heating begins in northern EC, and the local EAQ was gradually poor. Furthermore, the climate in EC is mainly controlled by the East Asian winter monsoon in winter, which results in a strong vertical temperature inversion layer, making air pollutants difficult to spread. Therefore, the AQI was relatively high in winter. The above results are consistent with previous research [49]. Moreover, as the largest fraction of energy consumption is in the Beijing–Tianjin–Hebei region [50,51], the highest AQI of each season was mainly distributed in the North China Plain. In contrast, because of being large and sparsely populated, and its animal husbandry production mode, the AQI was relatively low in the central and eastern Inner Mongolia border regions (Figure 2a). The distributions of AQI were relatively low in southeast coastal regions as well, which have abundant rainfall and high air humidity all year round. There was a strong anti-correlation between air humidity and AQI values [29]. Compared with 2015, the AQI in spring and summer did not change significantly in 2016 (Figure 2b). In autumn, the AQI distributed in Hebei, Shanxi, and Shaanxi increased significantly, while it decreased in Northeast China. In winter, the distributions of AQI showed a significant increase in Hebei, Shanxi, and parts of southern EC. In 2017, the distributions of AQI increased in the summer in Shanxi and Northern EC relative to 2015 (Figure 2c). In winter, the AQI distribution in Hebei decreased significantly, while it increased significantly in southern EC. In 2018, air pollution generally increased in northern EC from the spring with reference to 2015 (Figure 2d), especially in the central and northern regions of Inner Mongolia. The distributions of AQI also increased significantly in Hebei, Shanxi, and central-northern Inner Mongolia in summer. In autumn, air quality improved significantly in the northeast and the North China Plain, which was the most polluted area in 2015. In winter, the EAQ in EC improved overall, and the distributions of AQI increased only in the central part of Shanxi Province.

In recent years, with the strengthening of air pollution governance efforts, the air quality in most areas of EC has been improving [52,53]. The concentration of SO_2_, CO and NO_2_, decreased year by year over the period of 2015–2018 (figures not shown), especially the SO_2_ concentration decreased significantly in EC. This indicates that the strict control of SO_2_ industrial emissions has been effective [49]. Although the concentration of particulate matter (PM_2.5_ and PM_10_) in EC has decreased slightly since 2015, it was still the primary pollutant in spring, autumn, and winter during 2015–2018 (high number of days in these seasons). Compared with 2015, the concentration of PM_2.5_ significantly increased in Hebei and Shanxi in the winter of 2016, and then decreased year-on-year in 2017 and 2018. The PM_10_ concentration in each season has been effectively controlled in heavy industrial regions, such as Beijing–Tianjin–Hebei, since 2016, but there was an increase in Inner Mongolia, Shanxi, and the southern regions in spring, autumn, and winter compared to 2015. However, O_3_ concentration was significantly increasing year by year in most areas of EC during 2015–2018, and has become the most important conventional air pollutant, which has aroused concern among scholars [17]. Especially in spring and in summer, due to the number of polluted days seriously exceeding the standard, O_3_ has become the primary ambient air pollutant in EC (figures not shown). To sum up, the spatial characteristics of AQI and air pollutants in EC had different manifestations in each year from 2015 to 2018. The concentrations of SO_2_, CO, and NO_2_ showed overall decreases in most areas of EC, and particulate matters (PM_2.5_ and PM_10_) and O_3_ are still the main air pollutants in EC.

## 4. The Major Modes of the AQI Distribution

It can be seen from the previous section, that the seasonal mean states of AQI show some differences year by year. Would this change in the mean state affect the monthly variations? To address this question, EOF analysis was applied to the monthly AQI in EC over the period of 2015–2018. The first two EOF modes were statistically distinguished from the rest of the EOF modes based on the significant test proposed by North et al. [54].

**Figure 3:**
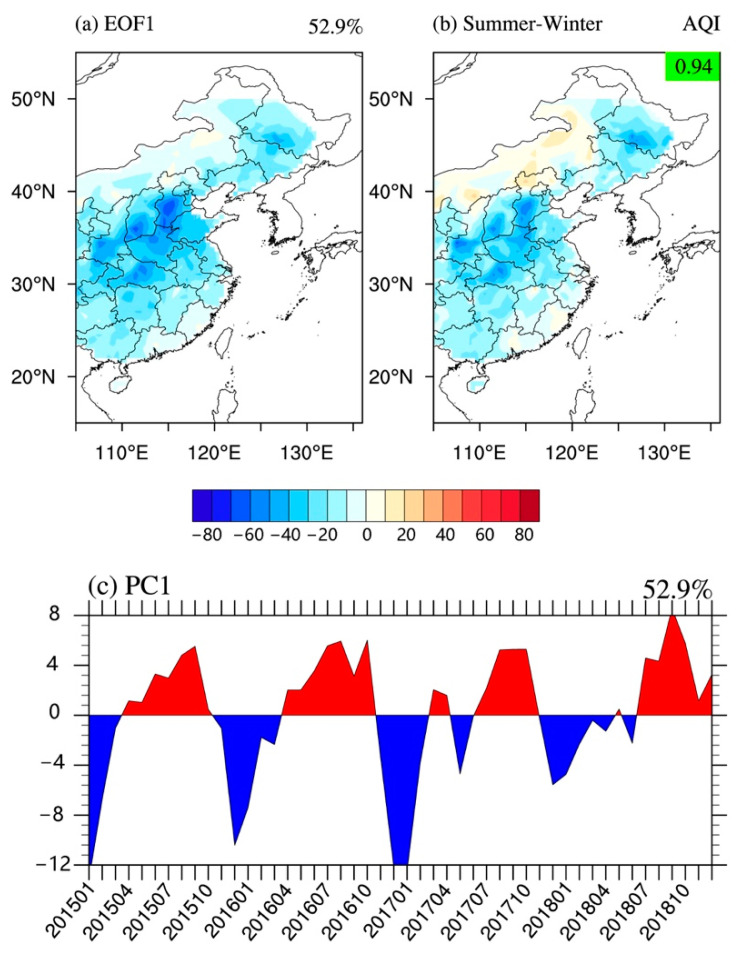
The leading EOF mode of the AQI variation in 2015–2018: (**a**) spatial structure (EOF1), (**c**) the corresponding principal component (PC1), and (**b**) spatial AQI distribution difference in the summer half year minus in winter half year. The fraction on the left top of panel (**a**,**c**) denotes the fractional explained variances of EOF1. The spatial correlation coefficients between (**a**,**b**) are shown at the top right corner of panel (**b**).

Figure 3 shows the spatial pattern of the leading EOF (EOF1) of the AQI in EC (Figure 3a) and the corresponding PC time series (PC1) for 2015–2018 (Figure 3c). The fractional variance explained by the EOF1 was 52.9%, accounting for more than half of the total variance, which represents a robust mode of spatial and temporal variabilities of the AQI in EC. The spatial pattern of EOF1 was characterized by a uniform structure throughout EC (Figure 3a), which exhibited a relatively large variation in the northern plains of China. The PC1 shown in Figure 3c indicates an obvious annual cycle variation from 2015 to 2018, which means that EAQ was better in summer and worse in winter in EC, consistent with the previous research results [55,56]. Therefore, the EOF1 of the AQI distribution in EC suggests an anti-correlation between winter and summer, which refers to this case as the “winter–summer” mode. Furthermore, the spatial pattern of the mean AQI in the summer half-year (May-September) minus that in the winter half-year (November-next March) was consistent with the spatial pattern of EOF1 during 2015–2018 (spatial correlation coefficient (CC) was 0.94, *p* < 0.01). Likewise, the same procedure of EOF analysis was applied to the six conventional air pollutants (figures not shown). The EOF1 of each air pollutant was similar to the EOF1 of AQI over EC (except for the leading mode of O_3_, which had a significant anti-correlation with the EOF1 of AQI). This means that the “winter–summer” mode is a common feature of the variations in each air pollutant in EC.

**Figure 4:**
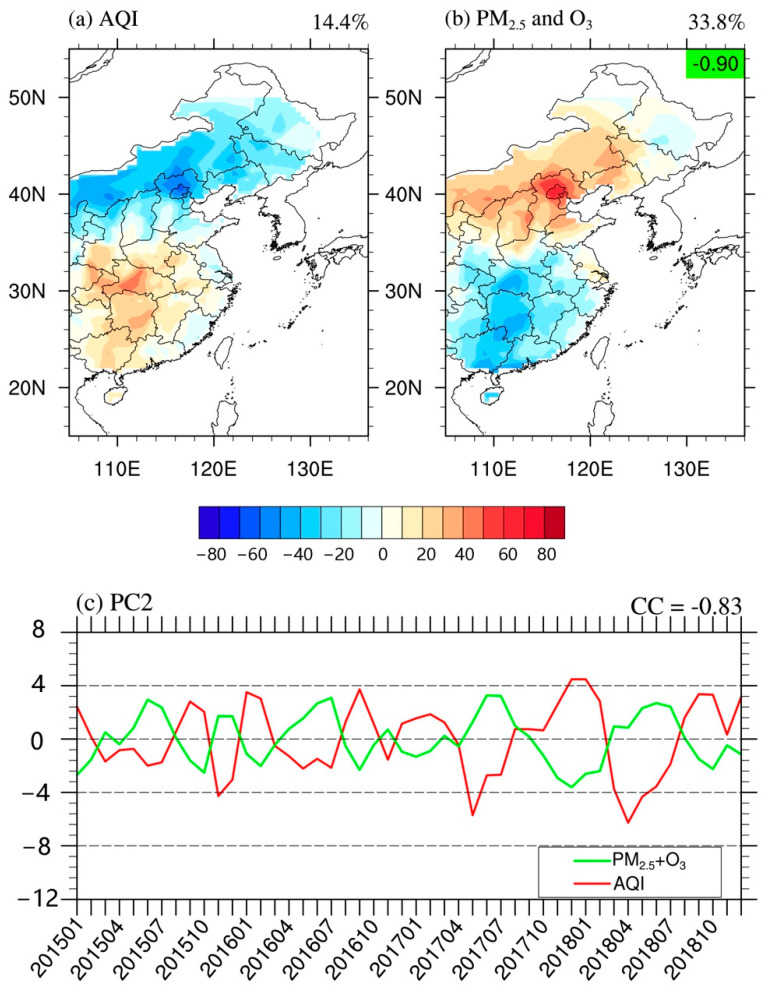
Comparisons of the spatial structures of the second EOF mode (EOF2) of (**a**) AQI and (**b**) ensemble mean of IAQIs of PM_2.5_ and O_3_, and their corresponding principal components (PC2, (**c**)). The red line indicates the PC2 for AQI, while the green line represents the PC2 for IAQI of the ensemble mean of PM_2.5_ and O_3_. The fraction on the top left of panel (**a**,**b**) denotes the fractional explained variances. The spatial correlation coefficients between (**a**,**b**) are shown at the top right corner of panel (**b**). The correlation coefficient between the two PC2s is drawn on the top right of panel (**c**).

**Figure 5:**
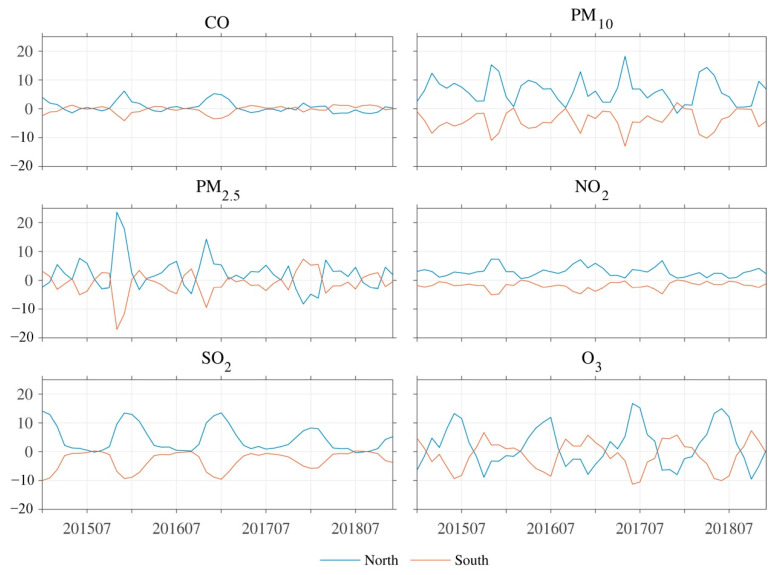
Monthly mean individual AQI anomalies of CO, PM_10_, PM_2.5_, NO_2_, SO_2_, and O_3_ in northern (blue lines) and southern (orange lines) Eastern China from 2015–2018.

**Figure 6:**
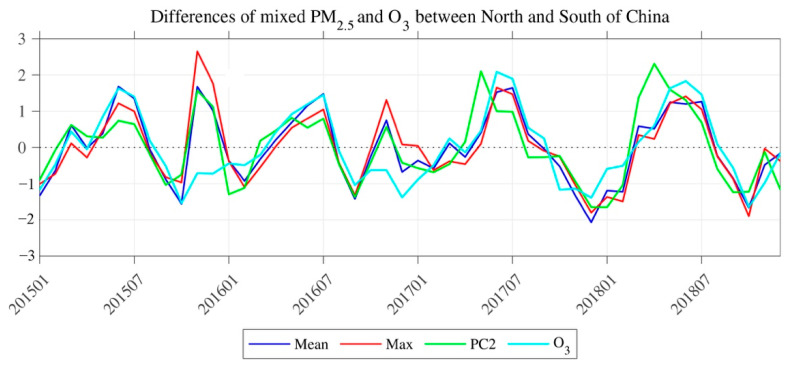
Normalized time series of AQI PC2 (green line), average differences of O_3_ IAQI (cyan line), ensemble mean of PM_2.5_ and O_3_ IAQI (blue line), and maximum of the PM_2.5_ and O_3_ IAQI (red line) between the south and north of Eastern China from 2015 to 2018.

The spatial structure of the second mode of AQI (EOF2) in EC is a north–south dipole pattern, characterized by the EAQ “north good (poor) and south poor (good)” (Figure 4a). The explained variance of EOF2 was 14.4%. The corresponding PC time series of EOF2 (PC2) was not significantly characterized by cycles and trends (Figure 4c). However, since the spring of 2017, the amplitude of PC2 intensity has significantly increased. By calculating the regional average IAQI in northern and southern EC (with 35° N as the boundary), respectively, the IAQIs of the six air pollutants all showed significant anti-correlations between northern and southern EC (CCs < −0.99, *p* < 0.01). Relative to EC-average IAQIs of CO, PM_10_, SO_2_, and NO_2_, the IAQIs in each month showed negative values over the southern EC and positive values over the northern EC (Figure 5). This indicates that these air pollutants over northern EC are always greater than those over southern EC. However, the IAQI of PM_2.5_ and O_3_ averaged over southern and northern EC in each month showed alternating positive and negative changes relative to these averages over EC (Figure 5). Therefore, the EOF2 of AQI was probably caused by the combined effect of PM_2.5_ and O_3_. By comparing the difference between the PC2 and the IAQIs (the average values over the northern EC minus the average values over the southern EC) of O_3_ and PM_2.5_, respectively (Figure 6), the changes of O_3_ during 2015–2018 follow that of the PC2 closely (CC = −0.58, *p* < 0.01). By combining the changes of PM_2.5_, the CC with PC2 was significantly increased (CC = −0.83, *p* < 0.01). Furthermore, the mean and standard deviation of O_3_ concentration over EC has shown an increased intensity since 2017, especially in summer. This indicates that O_3_ has become more serious and widespread in EC during 2015–2018, which is also in line with the characteristics of the increased amplitude of PC2 since the spring of 2017. The EOF2 of the combined mean IAQIs of PM_2.5_ and O_3_ (Figure 4b) exhibited an obvious north–south dipole pattern (CC = −0.90, *p* < 0.01), and the explained variance was 20.40%, which is slightly lower than its EOF1 explained variance (31.4%, figure not shown).

In summary, the combined effect of O_3_ and PM_2.5_ is the reason for the AQI north–south dipole pattern during 2015–2018. In recent years, although the governance of PM_10_ has had remarkable achievements, O_3_ and PM_2.5_ have gradually become important air pollutants that affect EAQ in EC. The PM_2.5_ and O_3_ of atmospheric composite pollution, and the north–south dipole pattern of AQI may become more and more serious in EC in the future.

## 5. The Relationship between Climatic Conditions and the Major Modes of AQI

To understand the relationship between climatic conditions and the major modes of AQI in EC, the climatic element fields were regressed onto the PCs of AQI during 2015–2018 for discussion. The residual terms of the regression were needed.

**Figure 7:**
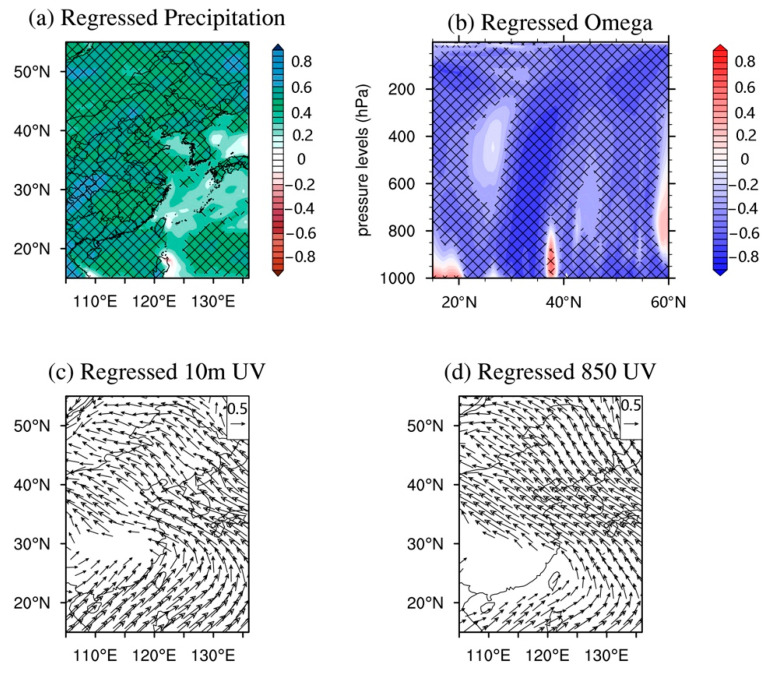
Regressed precipitation (**a**), vertical velocity (**b**), surface wind at 10 m (**c**), and 850 hPa wind (**d**) on the AQI PC1. The black crosses in panels (**a**,**b**) represent regions with regression significant at 99% confidence level. Shown in panel (**c**,**d**) are only the wind vectors that are significant above a 99% confidence level.

The EOF1 of AQI in EC refers to as the “summer–winter” mode (Figure 3a). Previous studies have shown that autumn and winter were heating seasons in northern China, and the emission of air pollutants in the north increased significantly in these seasons [49]. This means that human activities have made important contributions to this mode. However, the impact of climatic conditions in EC cannot be ignored. Figure 7a shows the PC1 of AQI in EC associated (regressed) with precipitation from 2015 to 2018. The spatial structure is characterized by a nearly uniform increase (or decrease) of precipitation across the EC (the black cross means an interval at a 99% confidence level, which is used hereafter). The interannual cycle of PC1 was more consistent with cycles of temperature and precipitation in EC, indicating that the monsoonal climate also has some impact on the EAQ. That is, in summer precipitation increases significantly, which has a strong capability for air purification. The same pattern is also reflected in vertical convection (Figure 7b). The “summer–winter” mode of AQI and the strength of convection change conversely, and significantly. This means that when the AQI decreases (increases) in summer, the atmospheric vertical motion in latitude increases (decreases). On the contrary, the thermal inversion layer is generally strengthened (weakened) in winter in EC, and the atmospheric vertical motion is reduced (enhanced). Furthermore, the AQI change in the mid-latitude region (30–40° N) (the North China Plain, Figure 3a) is more sensitive to the strengthening of vertical convection, which is in line with the previous studies [20,56]. Atmospheric circulation is also one of the main climatic factors affecting the AQI. In summer, the climate in southern EC is affected by warm and humid air currents in the Indian Ocean, and the climate in northern EC is affected by water vapor transport from the nearby Pacific Ocean (Figure 7c,d). The enhanced land–sea thermal contrast may bring more water vapor from the Pacific to EC, especially in coastal areas, and the EAQ is relatively good. Conversely, with the dry and cold air in northern China passing through the EC in winter, the thermal inversion layer becomes thicker, and the vertical convection is weak, then the precipitation is reduced, and results in the EAQ being relatively poor in winter. Similarly, the low-level wind field also shows the typical characteristics of a sea­–land breeze (Figure 7d). In summary, the surface and the low-level wind fields can bring warm and humid air from nearby oceans into EC, and the increased water vapor can deposit and absorb air pollutants, which leads to lower AQI in EC in summer. In winter, northern EC begins the heating season with a significant increase in pollution, and the East Asian winter monsoon climatic condition causes strong northerly winds, which bring air pollutants from northern to southern EC. In addition, due to the continuous consumption of energy in winter in northern EC, the overall AQI values over the entirety of EC are relatively higher in winter.

**Figure 8:**
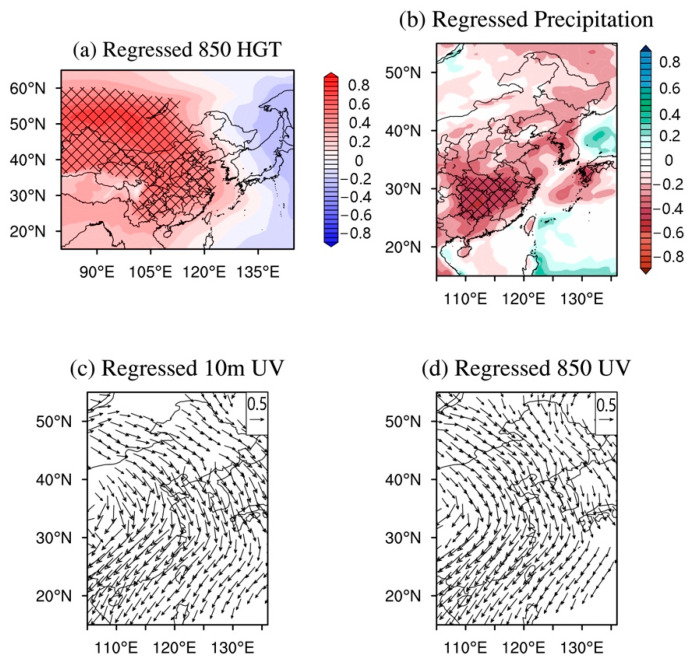
Regressed 850 hPa geopotential height (**a**), precipitation (**b**), surface wind at 10 m (**c**), and 850 hPa wind (**d**) on the AQI PC2. The black crosses in panel (**a**,**b**) represent the region with regression significant at a 99% confidence level, shown in panel (**c**,**d**) are only wind vectors that are significant above a 99% confidence level.

The spatial structure of AQI EOF2 in EC is characterized by a north–south dipole pattern (Figure 4a). The associations of PC2 and various climatic element fields indicate that the north–south dipole pattern of AQI is closely related to the intensity of the Mongolia–Siberian high (Figure 8a). The dipole pattern, i.e., the AQI in the south is high and that in the north is low, usually occurs in autumn and winter (Figure 4c). It is usually associated with the gradual increase of the Mongolia–Siberian high (Figure 8a), which causes stronger cold airflow from north to south, bringing air pollutants from northern EC to southern EC (Figure 8c,d). Meanwhile, the climate in southern EC is not in the rainy season and the air purification capacity is relatively weak (Figure 8b). This would induce the pattern that the regional AQI is increased in southern EC, while being decreased in northern EC. On the contrary, the dipole pattern, i.e., the AQI in the north is high and that in the south is low, mostly happens in spring and summer (Figure 4c). In these seasons, the temperature begins to increase in EC and precipitation generally begins and continues in southern EC. The land–sea thermal gradient progressively increases between the temperature over the land in EC and the northern Indian Ocean. The low-level and surface winds are predominantly south-western (Figure 8c,d). Summer Asian monsoon precipitation lands in southern EC from May (Figure 8b), and the air purification capacity over southern EC is strengthened. At this time, the monsoon precipitation in northern EC has not yet arrived (generally concentrated in July and August) and the Mongolia–Siberian high is gradually weakening and disappearing. The seasonal northerly wind has decreased. Therefore, the AQI in the southern EC has decreased and that in the northern EC has increased. Therefore, AQI EOF2 is manifested as the influence of climate on the migration and purification of air pollutants. The AQI in southern EC is closely related to precipitation changes in spring and summer, and the AQI in northern EC is significantly related to the intensity of the Mongolia–Siberian high in autumn and winter.

## 6. Conclusions

EAQ is one of the most concerning environmental issues in China, and understanding the characteristics of large-scale EAQ is essential for the implementation of the joint prevention and control of regional air pollution. In this study, we used the concentrations of six major air pollutants derived from about 1300 environmental monitoring stations in EC (from January 2015 to December 2018) to analyze the monthly average AQI according to national standards (GB3095-2012). The main seasonal spatial and temporal characteristics of the EAQ and its relationship with climatic conditions are as follows:

1. Generally, the AQI decreased in each season in EC from 2015 to 2018, but in spring and summer, the AQI distributions in north-central Inner Mongolia and Shanxi Province increased from 2015–2018.
2. From 2015 to 2018, the leading AQI mode in EC was characterized by the “summer and winter” mode, which was uniform across the EC. The AQI in EC generally increased during the winter half-year, and decreased during the summer half-year. In addition to human activities, climatic conditions, such as the East Asian monsoon, may play an important role in changes in EAQ patterns over the EC.
3. From 2015 to 2018, the second EOF mode of AQI in EC shows the characteristics of a north–south dipole pattern. That is, as the AQI in southern EC increased (decreases), it decreased (increases) in northern EC. PM_2.5_ and O_3_ are the main air pollutants associated with the AQI dipole pattern. In terms of a mechanism of climate conditions, the AQI in southern EC is closely related to precipitation changes in spring and summer, and the AQI in northern EC is significantly related to the intensity of the Mongolia–Siberian high in autumn and winter.

Air pollution is commonly affected by human activities and climatic factors. Human activities are the intrinsic factors, and climatic/meteorological conditions are the extrinsic factors. This was the point of departure for the present study. Based on the analysis of observational data, this study also shows that seasonal climatic conditions are one of the main factors affecting EAQ in EC, especially in the spreading and migration of air pollutants. However, due to the short period of EAQ monitoring in China, the data are not extensive enough to perform reasonable analyses for large-scale AQI changes on annual to decadal timescales. Nonetheless, this study can provide a reference for understanding the relationship between climatic conditions and EAQ.

## Figures and Tables

**Table 1:** Information on ERA-interim reanalysis used in this study.

Climatic Elements	Spatial Resolution	Vertical Layer	Units
2 m temperature	0.5° × 0.5°	1	K
Geopotential height field	0.5° × 0.5°	37	m^2^/s^2^
Precipitation	0.5° × 0.5°	1	m
Atmospheric vertical motion	0.5° × 0.5°	37	Pa/s
10 m wind field	0.5° × 0.5°	1	m/s
Wind field	0.5° × 0.5°	37	m/s

**Table 2:** Statistics of effective monitoring stations and the ratio of the total monitoring stations for CO, NO_2_, SO_2_, 8 h O_3_, PM_10_ and PM_2.5_, respectively.

Year	SO_2_	NO_2_	CO	O_3_	PM_10_	PM_2.5_
2015	1392 (86.9%)	1386 (86.5%)	1387 (86.6%)	1387 (86.6%)	1368 (85.4%)	1387 (86.6%)
2016	1367 (85.3%)	1365 (85.2%)	1367 (85.3%)	1381 (85.6%)	1359 (85.8%)	1363 (85.1%)
2017	1310 (81.8%)	1310 (81.8%)	1308 (81.6%)	1310 (81.8%)	1302 (81.3%)	1308 (81.6%)
2018	1343 (83.8%)	1343 (83.8%)	1342 (83.8%)	1344 (83.9%)	1314 (82.0%)	1340 (83.6%)

**Table 3:** Individual air quality index (IAQI) and corresponding concentration limit of pollution items (HJ633-2012).

IAQI	SO_2_ (μg/m^3^)	NO_2_ (μg/m^3^)	CO (mg/m^3^)	PM_10_ (μg/m^3^)	PM_2.5_ (μg/m^3^)	O_3_ (μg/m^3^)
0	0	0	0	0	0	0
50	50	40	2	50	35	100
100	150	80	4	150	75	160
150	475	180	14	250	115	215
200	800	280	24	350	150	265
300	1600	565	36	420	250	800
400	2100	750	48	500	350	1000
500	2620	940	60	600	500	1200

## Data Availability

Publicly available datasets were analyzed in this study. This data can be found here: Air pollution data (http://113.108.142.147:20035/emcpublish/, accessed on 1 February 2021), climate data (https://www.ecmwf.int/en/forecasts/datasets/reanalysis-datasets/era-interim, accessed on 1 May 2019).
